# Associations between obstructive sleep apnea risk and urinary incontinence: Insights from a nationally representative survey

**DOI:** 10.1371/journal.pone.0312869

**Published:** 2024-11-01

**Authors:** Bo Li, Feng Li, Xi Xie, Chenhui Xiang, Meilin Li

**Affiliations:** 1 Department of Urology, Clinical Medical College and The First Affiliated Hospital of Chengdu Medical College, Chengdu Medical College, Chengdu, People’s Republic of China; 2 Department of Gynecology, Clinical Medical College and The First Affiliated Hospital of Chengdu Medical College, Chengdu Medical College, Chengdu, People’s Republic of China; Kyung Hee University School of Medicine, REPUBLIC OF KOREA

## Abstract

Obstructive sleep apnea (OSA) and urinary incontinence (UI) are two prevalent health conditions with significant impacts on individuals’ quality of life. Although they appear distinct in nature, a growing body of evidence suggests a potential interrelationship between these conditions. Our objective was to explore the association between the risk of OSA and the occurrence of UI within a nationally representative sample of U.S. adults. Utilizing cross-sectional data from the National Health and Nutrition Examination Survey spanning the years 2015 to 2020, we conducted an analysis on a sample comprising 8,647 adults who provided comprehensive self-reported information on both UI and sleep apnea symptoms. The included cohorts were analyzed based on their sex. We employed the Multivariate Apnea Prediction (MAP) index to evaluate the risk of OSA. Subgroup analyses were conducted, categorizing them according to different types of UI. The association between OSA risk and UI was estimated through multivariable binary logistic regression models. After adjusting for relevant confounders, our results revealed a positive correlation between OSA risk and UI in both males (OR = 5.68, 95% CI = 1.74–18.47) and females (OR = 5.99, 95% CI = 2.68–13.41). The subgroup analysis illustrates that an elevated risk of OSA heightens the likelihood of experiencing stress urinary incontinence (SUI), urge urinary incontinence (UUI), and mixed urinary incontinence (MUI) in both male and female populations. Our study findings imply that an elevated risk of OSA exacerbates the likelihood of UI, SUI, UUI and MUI.

## Introduction

Obstructive sleep apnea (OSA) has garnered acknowledgment as a prevalent sleep disorder marked by recurrent occurrences of partial or total upper airway obstruction during slumber [1]. The estimated mean prevalence of OSA stands at 27.3% (range 9–86%) among males and 22.5% (3.7–63.7%) among females [2]. The resultant disrupted sleep patterns, intermittent hypoxia, and chronic sympathetic activation associated with OSA contribute to an array of cardiovascular [3], metabolic [4], and cognitive impairments [5]. While the core pathophysiology of OSA revolves around airway collapse and respiratory disturbances, the cascading effects of disrupted sleep on various physiological systems warrant a comprehensive investigation of potential downstream consequences.

The diagnosis of OSA mandates an all-encompassing sleep assessment conducted through polysomnography, within a closely supervised sleep laboratory [6]. The procedure is onerous, hence questionnaires and clinical predictive algorithms are frequently applied in clinical practice to pinpoint those individuals with an elevated propensity for OSA, thereby expediting subsequent clinical assessment [7]. Indeed, an effective tool for screening patients with OSA is the Multivariate Apnea Prediction (MAP) index [8]. This algorithm takes into account factors such as the frequency of sleep apnea symptoms, age, sex and body mass index (BMI) to furnish an estimation of the likelihood of OSA [8, 9].

Urinary incontinence (UI), delineated by the involuntary release of urine, exhibits a reported prevalence exceeding 10% among men surpassing the age of 60 [10], and surpassing 15% among women aged over 20 [11], with a propensity to escalate as the years advance [12]. UI can be further categorized into three distinct types: stress urinary incontinence (SUI), characterized by the inadvertent release of urine during activities that exert pressure on the bladder [13]; urge urinary incontinence (UUI), marked by an abrupt and compelling need to urinate resulting in involuntary leakage [14]; and mixed urinary incontinence (MUI), which presents as a combination of both stress and urge incontinence. The socio-economic burden of urinary incontinence is substantial, encompassing medical costs, reduced productivity, and diminished psychological well-being [15].

Recent research endeavors have uncovered intriguing associations between UI and OSA. Shared risk factors, such as obesity [16], raise the possibility of shared pathophysiological mechanisms. Sleep fragmentation, a hallmark of OSA, may disrupt neural, hormonal, and musculoskeletal processes pertinent to bladder function [17]. Chronic inflammation [5], a recognized mediator in both conditions, could further underscore their interconnectedness. Additionally, effective treatment of OSA, such as continuous positive airway pressure therapy, has demonstrated an impact on UI symptoms [18], raising questions about causal relationships and potential avenues for intervention.

UI and OSA are two prevalent and multifaceted medical conditions that significantly impact the quality of life and well-being of affected individuals. Although seemingly distinct in nature—one pertaining to bladder control and the other to respiratory function—a growing body of evidence suggests an intriguing interrelationship between these two conditions.

Consequently, we hypothesized that potential correlations could exist between OSA and UI. Hitherto, research regarding the correlation between OSA and UI remains a subject of contention. In consideration of this, we embarked on an analysis of the datasets from the National Health and Nutrition Examination Survey (NHANES) to ascertain the conceivable association between the risk of OSA and the incidence of UI.

## Materials and methods

The NHANES database stands as a substantial, all-encompassing multi-stage complex sampling survey initiative within the United States. Data were primarily procured through individual interviews with households, health assessments conducted at mobile screening facilities, and examination of laboratory specimens. Prior to inclusion in the NHANES database, all participants provided written informed consent and received approval from the National Center of Health Statistics Research Ethics Review Board. Our analysis covers 6 years of NHANES data——2015 to 2020 year. Individuals included in the study had complete symptoms of UI and OSA. Specifically, the following parameters were collected self-reported by questionnaire: age, sex, marital status (married or living with partner, living alone), race (Mexican American, non-Hispanic Asian, non-Hispanic black, non-Hispanic white, other Hispanic, other race), physical activity in recreational time (no, yes), education status (under high school, high school or equivalent, above high school), family poverty income ratio (PIR) (≤1.3, 1.3–3.5, ≥3.5), smoking status (never, former, now), body mass index (BMI) (normal weight <25, overweight 25–30, and obesity ≥30).

The MAP index serves as a screening instrument employed to forecast the likelihood of OSA in a population [8]. The MAP index combines self-reported sleep apnea symptoms with factors such as age, sex, and BMI to calculate a probability index (ranging from 0 to 1) for OSA. A score of 0 indicates the lowest risk of having OSA, while a score of 1 suggests the highest risk. Self-reported sleep apnea symptoms include "loud snoring," "snorting or gasping," and "cessation of breathing or choking or struggling for breath." Similar questions about sleep apnea symptoms are included in the NHANES database and to assess participants’ "obstructive sleep apnea risk" [4]. Low risk of OSA indicates MAP score of < 0.5, whereas high risk of OSA denotes MAP score ≥0.5 [8].

In the NHANES database, there are two questions related to UI: "During the past 12 months, have you leaked or lost control of even a small amount of urine with an activity like coughing, lifting, or exercise?" If the respondent answers "Yes," it is diagnosed as SUI. "During the past 12 months, have you leaked or lost control of even a small amount of urine with an urge or pressure to urinate and you couldn’t get to the toilet fast enough?" If the respondent answers "Yes," it is diagnosed as UUI. If a respondent answers "Yes" to both questions, it is diagnosed as MUI. If a respondent answers "Yes" to either question, it is diagnosed as UI.

All analyses were executed utilizing R Studio version 4.2.2. All the data have been weighted according to NHANES guidelines to account for sample weights. Continuous variables were computed as means ± standard deviations (SD), while categorical variables were tabulated as counts (percentages). Categorical variables are scrutinized employing the chi-square test, whereas continuous variables undergo analysis via the t-test to discern disparities between groups. Given that there may be differences in the mechanisms of UI between male and female, and there are noticeable differences in the prevalence of UI between sex, group analyses have been conducted based on sex. The association between risk of OSA and UI was estimated by multivariable binary logistics regression models with odds ratio (OR) and 95% confidence interval (CI). Moreover, a multivariable model was established to account for age, race, recreational physical activity, smoking status, and BMI. A bilateral *P*<0.05 is regarded as statistically significant.

## Results

### Characteristics of the included population

**Fig 1:**
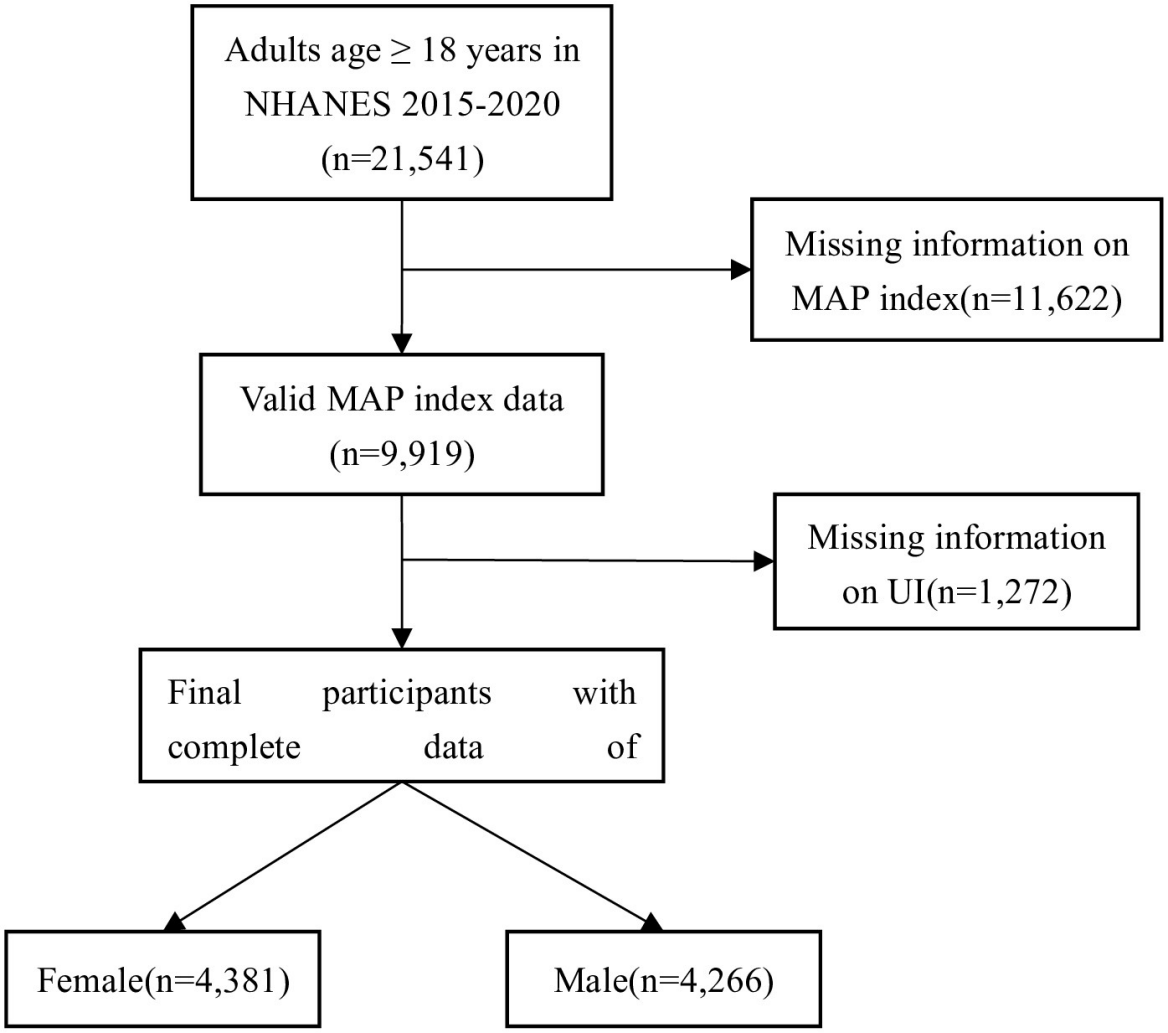
Flowchart detailing participant selection. NHANES, the National Health and Nutrition Examination Survey; MAP, multivariate apnea prediction; UI, urinary incontinence.

Our analysis encompassed four of NHANES data, spanning from 2015–2016, 20017–2018 and 2019–2020. In total, 21,541 individuals, aged 18 and above were interviewed and underwent relevant examinations in the NHANES database. Out of these participants, 9,919 individuals had complete records for the frequency of "snoring" and "snort or stop breathing." Among those with complete data on UI, which totaled 8,647 individuals, there were 4,381 females and 4,266 males (Fig 1). The basic characteristics of 4,381 females and 4,266 males were delineated within Table 1. The t-test and chi-square test found significant correlations between the following factors and female UI: age, race, BMI, physical activity in recreational time (P<0.05). Similarly, for male UI, significant associations were found with age, BMI, physical activity in recreational time(P<0.05). In the female population, the average MAP Score is 0.29±0.01. Among females with UI, the MAP score is significantly higher compared to the normal population (i.e., 0.34 vs. 0.21, p<0.001). In the male population, the average MAP score is 0.53±0.01, and among males with UI, the MAP score is significantly higher compared to the normal population (i.e., 0.65 vs. 0.51, p<0.001). Within the female demographic, the prevalence of High OSA risk stands at 19.53%. Among female individuals experiencing UI, the prevalence of High OSA risk is notably greater than that observed in the normal population (27.34% vs. 9.67%, p<0.001). Within the male demographic, the prevalence of High OSA risk stands at 57.62%. Among males grappling with UI, the incidence of High OSA risk is markedly greater when compared to the normal (76.30% vs. 54.22%, p<0.001).

**Table 1:** Basic characteristics of the study population according to sex.

	Female				Male			
	total	normal	UI	*P* value	total	normal	UI	*P* value
Age	48.20±0.51	42.47±0.56	52.73±0.56	< 0.001	47.15±0.44	45.05±0.37	58.70±0.88	< 0.001
Race				< 0.001				0.1
mexican american	692(15.8)	309(8.62)	383(7.69)		632(14.81)	527(9.61)	105(7.36)	
non-hispanic asian	539(12.3)	307(7.25)	232(3.96)		530(12.42)	477(5.54)	53(3.24)	
non-hispanic black	972(22.19)	477(13.35)	495(9.90)		917(21.5)	704(9.92)	213(11.82)	
non-hispanic white	1478(33.74)	559(58.87)	919(69.44)		1519(35.61)	1202(63.74)	317(66.27)	
other hispanic	524(11.96)	280(8.45)	244(4.72)		458(10.74)	360(6.66)	98(6.62)	
other race	176(4.02)	85(3.46)	91(4.29)		210(4.92)	170(4.53)	40(4.69)	
Physical activity in recreational time				0.002				< 0.001
no	3456(78.89)	1505(69.82)	1951(78.80)		3027(70.96)	2346(64.31)	681(79.13)	
yes	925(21.11)	512(30.18)	413(21.20)		1239(29.04)	1094(35.69)	145(20.87)	
Marital status								
married or living with partner	2503(62.67)	1143(61.60)	1360(63.55)	0.32	2879(69.18)	2305(68.62)	574(72.30)	0.17
living alone	1875(37.31)	872(38.40)	1003(36.45)		1385(30.81)	1133(31.38)	252(27.70)	
Education status				0.41				0.09
under high school	826(18.85)	364(9.99)	462(11.17)		923(21.64)	706(12.03)	217(16.07)	
high school or equivalent	935(21.34)	418(21.92)	517(22.85)		1032(24.19)	839(25.94)	193(24.85)	
above high school	2620(59.8)	1235(68.09)	1385(65.98)		2311(54.17)	1895(62.03)	416(59.07)	
Smoking status				< 0.001				< 0.001
never	2995(68.39)	1480(70.96)	1515(61.36)		2024(47.48)	1728(51.06)	296(37.64)	
former	727(16.6)	256(14.44)	471(22.32)		1290(30.26)	953(29.55)	337(40.24)	
now	657(15)	279(14.60)	378(16.32)		949(22.26)	757(19.39)	192(22.12)	
BMI				< 0.001				0.03
≤25	1246(28.44)	751(40.76)	495(24.48)		1096(25.69)	923(25.16)	173(20.62)	
25–30	1199(27.37)	535(26.03)	664(27.29)		1535(35.98)	1250(35.02)	285(34.00)	
≥30	1936(44.19)	731(33.21)	1205(48.23)		1635(38.33)	1267(39.82)	368(45.38)	
PIR				0.92				0.23
≤1.3	1203(30.78)	557(21.10)	646(20.72)		1043(27.28)	830(17.68)	213(18.23)	
1.3–3.5	1556(39.82)	677(35.93)	879(36.68)		1598(41.8)	1262(35.63)	336(40.38)	
≥3.5	1149(29.4)	531(42.96)	618(42.60)		1182(30.92)	999(46.69)	183(41.39)	
MAP score	0.29±0.01	0.21±0.01	0.34±0.01	< 0.001	0.53±0.01	0.51±0.01	0.65±0.01	< 0.001
High OSA risk	921(19.53)	244(9.67)	677(27.34)	< 0.001	2542(57.62)	1894(54.22)	648(76.30)	< 0.001

UI, urinary incontinence; MAP, Multivariate Apnea Prediction; BMI, body mass index; PIR, family poverty income ratio; OSA, obstructive sleep apnea.

### Associations of OSA risk with UI

In Table 2, the outcomes of the binary logistic regression analyses reveal the relationship between the MAP score, High OSA risk, and UI within the presented models. In unadjusted models, it was observed that for each unit increase in the log-transformed MAP score, the association with UI was positively significant for both females (OR = 19.47, 95% CI = 10.93–34.67) and males (OR = 14.83, 95% CI = 8.42–26.13). Similarly, in a parallel fashion, the association remained statistically significant in the multivariable model, which incorporated adjustments for age, race, recreational physical activity, smoking status, and BMI variables (female: OR = 5.99, 95% CI = 2.68–13.41; male: OR = 5.68, 95% CI = 1.74–18.47). In unadjusted models, the probability of experiencing UI is notably greater in the high OSA risk group when compared to the low OSA risk group (female: OR = 3.52, 95% CI = 2.66–4.64; male: OR = 2.72, 95% CI = 2.04–3.63). In multivariable models, the likelihood of UI in females with high OSA risk is still significantly higher than the low OSA risk (OR = 1.92, 95% CI = 1.53–2.41). However, in multivariable models for males, the likelihood of UI in the High OSA risk is not significantly different from the low OSA risk (p = 0.19).

**Table 2:** Association between risk of OSA and UI among US adults.

	Female		Male	
MAP index	unadjusted model		unadjusted model	
	OR 95% CI	*P* value	OR 95% CI	*P* value
	19.47(10.93,34.67)	<0.001	14.83(8.42,26.13)	<0.001
MAP index	multivariable model		multivariable model	
	OR 95% CI	*P* value	OR 95% CI	*P* value
	5.99(2.68,13.41)	<0.001	5.68(1.74,18.47)	0.01
High OSA risk	unadjusted model		Unadjusted model	
	OR 95% CI	*P* value	OR 95% CI	*P* value
	3.52(2.66,4.64)	<0.001	2.72(2.04,3.63)	<0.001
High OSA risk	multivariable model		multivariable model	
	OR 95% CI	*P* value	OR 95% CI	*P* value
	1.92(1.53,2.41)	<0.001	1.32(0.86,2.01)	0.19

MAP, Multivariate Apnea Prediction; OSA, obstructive sleep apnea; OR, odds ratio; CI, confidence interval.

### Subgroup analysis

The outcomes of the subgroup analysis are evident within Table 3. Within the female demographic, it was noted that an escalation in the MAP score significantly elevated the susceptibility to SUI (OR = 8.43, 95% CI = 4.98–14.28), UUI (OR = 17.26, 95% CI = 11.18–26.63), and MUI (OR = 19.52, 95% CI = 13.59–28.02). After adjusting for age, race, physical activity in recreational time, smoking status, and BMI in females, we still found that an increase in MAP score markedly heightened the risk of SUI (OR = 3.73, 95% CI = 1.86–7.51), UUI (OR = 5.19, 95% CI = 2.74–9.82), and MUI (OR = 5.93, 95% CI = 2.98–11.82). Similar results were observed in the male population. In males, we found that an increase in MAP score significantly escalated the risk of SUI (OR = 13.74, 95% CI = 4.70–40.18), UUI (OR = 19.27, 95% CI = 11.78–31.53), and MUI (OR = 138.04, 95% CI = 134.32–555.29). In the multivariable model for males, we found that an increase in MAP score still significantly augmented the risk of SUI (OR = 7.24, 95% CI = 1.23–42.66), UUI (OR = 7.08, 95% CI = 2.23–22.49), and MUI (OR = 64.36, 95% CI = 9.59–431.88). We discovered that the high OSA risk group exhibited a greater probability of developing SUI (female: OR = 2.35, 95% CI = 1.83–3.03; male: OR = 2.58, 95% CI = 1.67–3.98), UUI (female: OR = 2.97, 95% CI = 2.39–3.70; male: OR = 3.04, 95% CI = 2.28–4.07), and MUI (female: OR = 2.91, 95% CI = 2.38–3.56; male: OR = 7.12, 95% CI = 3.69–13.73) in comparison to the low OSA risk group. However, these results differ between the male and female populations after adjusting for age, race, BMI, smoking status, and physical activity in recreational time. In multivariable models, females within the high OSA risk category display an increased likelihood of developing SUI (OR = 1.48, 95% CI = 1.18–1.85), UUI (OR = 1.48, 95% CI = 1.17–1.87), and MUI (OR = 1.34, 95% CI = 1.07–1.69) in contrast to the low OSA risk group. In multivariable models for males, those within the high OSA risk category exhibit an increased likelihood of developing MUI (OR = 3.59, 95% CI = 1.72–7.50) when compared to the low OSA risk group.

**Table 3:** Association between risk of OSA and different types of UI among US adults.

	Female						Male					
MAP index	unadjusted model						unadjusted model					
	MUI		SUI		UUI		MUI		SUI		UUI	
	OR	*P*	OR	*P*	OR	*P*	OR	*P*	OR	*P*	OR	*P*
	95% CI		95% CI		95% CI		95% CI		95% CI		95% CI	
	19.52	<0.001	8.43	<0.001	17.26	<0.001	138.04	<0.001	13.74	<0.001	19.27	<0.001
	(13.59,28.02)		(4.98,14.28)		(11.18,26.63)		(34.32,555.29)		(4.70,40.18)		(11.78,31.53)	
MAP index	multivariable model						multivariable model					
	MUI		SUI		UUI		MUI		SUI		UUI	
	OR	*P*	OR	*P*	OR	*P*	OR	*P*	OR	*P*	OR	*P*
	95% CI		95% CI		95% CI		95% CI		95% CI		95% CI	
	5.93	<0.001	3.73	<0.001	5.19	<0.001	64.36	<0.001	7.24	0.03	7.08	0.002
	(2.98,11.82)		(1.86,7.51)		(2.74,9.82)		(9.59,431.88)		(1.23,42.66)		(2.23,22.49)	
High OSA risk	unadjusted model						unadjusted model					
	MUI		SUI		UUI		MUI		SUI		UUI	
	OR	*P*	OR	*P*	OR	*P*	OR	*P*	OR	*P*	OR	*P*
	95% CI		95% CI		95% CI		95% CI		95% CI		95% CI	
	2.91	<0.001	2.35	<0.001	2.97	<0.001	7.12	<0.001	2.58	<0.001	3.04	<0.001
	(2.38,3.56)		(1.83,3.03)		(2.39,3.70)		(3.69,13.73)		(1.67,3.98)		(2.28,4.07)	
High OSA risk	multivariable model						multivariable model					
	MUI		SUI		UUI		MUI		SUI		UUI	
	OR	*P*	OR	*P*	OR	*P*	OR	*P*	OR	*P*	OR	*P*
	95% CI		95% CI		95% CI		95% CI		95% CI		95% CI	
	1.34	0.02	1.48	0.002	1.48	0.002	3.59	0.002	1.50	0.2	1.41	0.12
	(1.07,1.69)		(1.18,1.85)		(1.17,1.87)		(1.72,7.50)		(0.79,2.83)		(0.90,2.19)	

MAP, Multivariate Apnea Prediction; OSA, obstructive sleep apnea; OR, odds ratio; CI, confidence interval; SUI, stress urinary incontinence; UUI, urge urinary incontinence; MUI, mixed urinary incontinence.

## Discussion

The current cross-sectional data from NHANES, demonstrates a positive correlation between risk of OSA and UI in ordinary adults. Our study found that an increase of one unit in log-transformed in risk of OSA among participants may be linked to an increased probability of experiencing UI. Even after accounting for various variables, an increase in the risk of OSA still shows a higher incidence of UI. And individuals at a high risk of OSA were more prone to experiencing UI. We observed that after adjusting for multiple variables, an increase in the susceptibility to OSA in significantly raised the risk of SUI, UUI and MUI occurrences both males and females. We found that in both males and females, the high OSA risk was more likely to develop SUI, UUI, and MUI than the low OSA risk. After adjusting for relevant variables, we found that in females, the group with a heightened susceptibility to OSA exhibits a greater probability of experiencing SUI, UUI, and MUI, while in males, the group at a heightened risk of OSA solely demonstrates an elevated probability of experiencing MUI.

Previous studies have suggested a correlation between OSA and lower urinary tract symptoms (LUTS). Currently, research regarding the correlation between OSA and UI is primarily based on small-sample retrospective studies, and there is debate among different studies on this matter. Kemmer H et al. indicated that in male patients, those individuals with moderate and severe OSA exhibited considerably elevated scores in the overactive bladder symptom score compared to patients with mild OSA, and severe patients with OSA were also at a heightened likelihood of experiencing daytime urgency symptoms as opposed to those with mild OSA [19]. However, Tuncer M et al. argued that in relatively young adult male patients with OSA, the prevalence of overactive bladder (OAB) and UUI was not increased. Additionally, a higher grading of OSA does not necessarily implied a higher prevalence of OAB [20]. Lowenstein L et al. found that in female patients, OSA was highly prevalent in patients with OAB, including those with pathological nocturia [21]. Yilmaz et al. provided further evidence of the link between OSA and LUTS, and indicated that OSA may play a role in the onset of LUTS by causing hypoxia [22].

The pathophysiology of OSA and UI is still not fully understood; in fact, it is likely the result of multiple factors interacting together. Lüdemann P et al. found that recurrent intermittent hypoxia is an independent risk factor for peripheral nerve axonal damage [23]. Nerve damage is a major cause of LUTS in patients with diabetes [24] and stroke [25], including urinary frequency and OAB symptoms. Yilmaz Z et al. believed that ischemic nerve damage caused by hypoxia may be the main cause of frequent urination in patients with OSA [22]. Both urinary continence and respiratory control involve complex neural pathways and autonomic nervous system regulation [26]. OSA can lead to intermittent hypoxia (low oxygen levels), which might affect the autonomic nervous system and potentially lead to changes in bladder function and continence control [27].

Inflammation may indeed hold a noteworthy position in the connection between OSA and UI. OSA is considered a low-grade chronic inflammatory state, and the existence of inflammation can be seen as a potential influencing factor in the pathophysiology and comorbidity [28]. Inflammatory factors may promote detrusor muscle activation and affect bladder sensory pathways [29]. Inflammation can indeed affect the connective tissues of the bladder and musculature of the pelvic floor, leading to a weakening of the pelvic floor support structures [30]. Furthermore, obesity serves as a prevalent risk factor for both OSA and UI. It can exacerbate OSA by increasing upper airway resistance [31]. Excess weight places added pressure on the bladder and pelvic support structures, potentially leading to urinary incontinence [32].

Here are the main strengths of this study. We considered the differences in UI between different sex and calculated the MAP score and high OSA risk between men and women, respectively. We have also explored the connection between the susceptibility to OSA, a heightened risk of OSA, and various forms of UI. Our analysis relies on a weighted sample that mirrors the distribution of the U.S. population, making our study broadly representative of the entire United States. To obtain more accurate results, we adjusted for key potential confounders. The limitations of this study encompass its cross-sectional design, precluding the establishment of causal relationships. Additionally, the lack of specific data of interest, such as polysomnography, limits the ability to objectively determine the presence of OSA and its severity.

## Conclusions

Our study findings imply that an elevated risk of OSA exacerbates the likelihood of UI, SUI, UUI and MUI, and strategies are essential to mitigate the risk of OSA and UI, SUI, UUI and MUI in different patient populations. Lowering the risk of OSA may reduce the risk of UI, thereby improving sleep quality and quality of life. Providers should consider assessing the severity of UI, if a patient’s screening discloses a heightened susceptibility to OSA, and vice versa. However, we need further research to clarify the underlying pathophysiological mechanisms.
